# Implementation of Omni-D Tele-Presence Robot Using Kalman Filter and Tricon Ultrasonic Sensors

**DOI:** 10.3390/s22103948

**Published:** 2022-05-23

**Authors:** Hassan Tariq, Muhammad Rashid, Asfa Javed, Muhammad Aaqib Riaz, Mohammed Sinky, Muhammad Yousuf Irfan Zia

**Affiliations:** 1 Department of Electrical Engineering, School of Engineering, University of Management and Technology (UMT), Lahore 54770, Pakistan; hassantariq@umt.edu.pk (H.T.); asfa.javed@umt.edu.pk (A.J.); f2018019075@umt.edu.pk (M.A.R.); 2 Department of Computer Engineering, Umm Al-Qura University, Makkah 21955, Saudi Arabia; mfelahi@uqu.edu.sa (M.R.); mhsinky@uqu.edu.sa (M.S.); 3 Telecommunications Engineering School, University of Malaga, 29010 Malaga, Spain

**Keywords:** omnidirectional robot, tele-presence robot, Kalman filter, Tricon ultrasonic sensors

## Abstract

The tele-presence robot is designed to set forth an economic solution to facilitate day-to-day normal activities in almost every field. There are several solutions to design tele-presence robots, e.g., Skype and team viewer, but it is pretty inappropriate to use Skype and extra hardware. Therefore, in this article, we have presented a robust implementation of the tele-presence robot. Our proposed omnidirectional tele-presence robot consists of (i) Tricon ultrasonic sensors, (ii) Kalman filter implementation and control, and (iii) integration of our developed WebRTC-based application with the omnidirectional tele-presence robot for video transmission. We present a new algorithm to encounter the sensor noise with the least number of sensors for the estimation of Kalman filter. We have simulated the complete model of robot in Simulink and Matlab for the tough paths and critical hurdles. The robot successfully prevents the collision and reaches the destination. The mean errors for the estimation of position and velocity are 5.77% and 2.04%. To achieve efficient and reliable video transmission, the quality factors such as resolution, encoding, average delay and throughput are resolved using the WebRTC along with the integration of the communication protocols. To protect the data transmission, we have implemented the SSL protocol and installed it on the server. We tested three different cases of video resolutions (i.e., 320×280 , 820×460 and 900×590 ) for the performance evaluation of the video transmission. For the highest resolution, our TPR takes 3.5 ms for the encoding, and the average delay is 2.70 ms with 900 × 590 pixels.

## 1. Introduction

In this modern era, robots dominate the top horizon of different branches of embedded systems. For example, in medical-related applications, the increasing number of people results in increasing difficulties for the operators (or physicians) to monitor and serve the severe patients. Thus, a virtual presence is a good alternative to aid patients in scenarios where face-to-face communication is not possible [1,2]. Video chats are very popular, but tele-presence gives the immense feeling of virtual presence from a distance, which enables the best power for decisions and resolves the complexity of the monitoring and daily routine work. With the increased population, the percentage of the population of elderly people is also increasing in the world. The World Health Organization (WHO) raises this concern that the population of people 60 years and above age is predicted to rise between 2000 and 2050. This means it will grow from 605 million to 2 billion and it will double from 11 to 22% [3]. The increase in total population and aged population has created economic, cultural and social challenges in the families, individuals, societies and the community globally.

To address the aforementioned issues, an attractive alternative is the use of a tele-presence robot. A tele-presence robot (TPR) is a holonomic remote-controlled, wheeled device that has wireless internet connectivity. Typically, the robot uses a tablet to provide video and audio capabilities. Moreover, the TPR contains the cameras, which allow the user(s) to enter a live conference, video chat, monitoring, etc. [4]. The TPR enables the people (or) groups of people to communicate with each other through the internet anywhere in the world. One of the solutions is to exploit the Internet protocols and the Skype cross-platforms for the design and development of tele-presence [5,6]. The solutions based on Skype have achieved some degrees of success in utilizing the software and hardware development. Nevertheless, the processing and handling time is high, as it exploits third-party plugins. In [7], a wheelchair-using tele-presence robot system is introduced for outdoor applications to support handicaps. Their system uses cellular data and depends on the Skype and Mac book mini. Their findings demonstrate that the use of combined hardware and software is not feasible for some applications. Additionally, the interfacing and synchronization of hardware and software result in further issues. Another method for tele-presence wheelchairs equipped with multiple cameras is proposed in [8] with an aid to provide effective assistance for the elderly and people with disabilities.

Another method is to use open-source APIs and integrate them into the system. WebRTC is the recent example that provides a free hand to modify, integrate and use in their way. This influences the user’s experience and the overall quality of the video calls. The use of WebRTC also offers numerous benefits for the service providers, i.e., ease of use, cost reduction, security, fast time to market, and simplified device integration. Moreover, with WebRTC, users can enjoy real-time communication services which are outside the traditional fixed and mobile device context [9]. Along with the video transmission, the control of the mobile robot is also an important step during the design and implementation of a tele-presence robot. Motivated by [7], the authors propose a video communication framework to improve the performance of a tele-presence wheelchair system further. Their mechanical framework is utilizing a non-holonomic robotic model which requires steering while turning the robot’s direction. Another method is to exploit the holonomic model, which can move from one point to another and can turn on a single point [10].

### 1.1. Motivation

The complete design consists of video transmission and control of the mobile robot. The implementation of webRTC is evolving with time and it still needs a performance improvement. Keeping this view, performance parameters, i.e., the average delay and throughput, require careful consideration during the implementation of a tele-presence robot. For different applications, the features discovered by researchers are increasing day by day. Some identical parameters include efficient and robust control with the highest possible degree of stability. Additionally, the control methodology, video quality, server security and efficiency are important parameters to characterize the TPR [11]. On the other hand, the collision-proof feature helps to secure from the damage. To improve the control, a state prediction and estimation mechanism should be integrated along with a collision-proof feature. Many applications require TPR such as monitoring in the medical field, conference meetings, invigilation, etc. Therefore, the medical-related applications are on the top to serve the betterment of humanity [12,13,14,15]. The TPR is also employed in the shopping malls for entertainment [16]. According to [17], the TPR is (also) well-suited for numerous applications in the COVID-19 pandemic where relatives and doctors can virtually meet the patients. Therefore, based on the scenario presented above, there is a real need to design and implement the tele-presence robot for medical-related applications. This is precisely the motivation for our work. In this paper, we propose a complete design of the robot that offers robustness in the video transmission as well as control of the mobile robot. The tele-presence robot consists of omnidirectional kinematics and exploits webRTC for video transmission. We propose a Tricon ultrasonic sensor algorithm to integrate a Kalman filter for the velocity and location estimation.

### 1.2. Related Work and Challenges

We classify the existing tele-presence based robot systems according to their utilized applications. Some tele-presence robots for educational activities are presented in [18,19,20]. Research practices describing the wheelchairs based on tele-presence robot systems are shown in [7,8]. Examples of some additional tele-presence robot systems are described in [10,21,22,23,24,25,26,27,28]. More insight details to these research practices are given below.

Tele-presence robots for educational activities [18,19,20]: Some interesting tele-presence robots are presented in [18,19] for teaching activities to facilitate the elderly instructors. This presents enough understanding to the students when they see the live activity on the screen. Moreover, this gives the concept of distance learning to improve the literacy rate, but the problem is that it must be under omnidirectional control because it will be used in educational institutes. An omnidirectional control determines the ability of the movement of TPR in any direction instantaneously from any starting position. In [20], a tele-presence robot for tutoring is presented to facilitate the students in the educational institutes. The robot contains a collision system to prevent damage to hardware. This includes a large screen and remote control of the movement for the lectures.

Wheelchairs based on tele-presence robot systems [7,8]: In [7], a wheelchair-using a tele-presence robot system is introduced for outdoor applications to support handicaps. Their system uses cellular data and depends on Skype and the mac book mini. Their findings demonstrate that the use of combined hardware and software is not feasible for some applications. Additionally, the interfacing and synchronization of hardware and software result in further issues. Similar to [7], a new method for tele-presence wheelchairs equipped with multiple cameras is proposed in [8] with an aid to provide effective assistance for the elderly and people with disabilities.

Some additional tele-presence robot systems [10,21,22,23,24,25,26,27,28]: In [21], a tele-presence robot is used as a medical doctor, which contains several beneficial features such as temperature pulse rate, etc. Additionally, a TPR is used to serve the patients in sequence to one another based on the instructions provided by the physician. An Arduino-based microcontroller unit (we termed MCU) is employed to provide instructions to the robot. The MCU is slow for processing when it has to receive the packets from the internet, which reduces its efficiency and eventually increases the command execution and propagation time. In [22], an assisted driving concept is introduced that typically provides collision avoidance. Moreover, their design avoids unnecessary movements that would lead to a collision. A virtual platform of an underwater manipulator mounted on a submersible vehicle is presented in [23]. They used a three-dimensional simulator “Webots” for teleoperation through a replica master arm.

A simple obstacle avoidance method is proposed in [24] for tele-presence robots to assist aged people living in remote areas. Their approach is appropriate for manually operated robot systems. More precisely, when an opening is not found, the robot stops and an operator is responsible for moving the robot by a remote operation to search for an opening. A low-cost telepresence robot with mechanical control of omni-wheels is presented in [10]. This provides them access to a wide range of directions. Their solution offers two different modes where the robot operates in a normal or autonomous mode. The authors in [25] designed a robot to include new expressive features such as Light-Emitting Diodes (LED), a robotic arm and some basic control functions to support disabled persons.

The maldistribution problem of physicians is resolved in [26] by using tele-presence robot systems. They noted that one limitation of the medical tele-presence robots system is the lack of medical features. Based on this observation, they developed a tele-presence system that includes a tele-presence robot (named, Akibot) integrated with medical devices such as an otoscope, stethoscope, and ultrasound probe. In [27], a novel method of state estimation using delayed sensor measurements of a TPR for real-time navigation is presented. An Augmented State Extended Kalman Filter (AS-EKF) is proposed to estimate the position of the robot. They tested their proposed algorithm in a real environment. They show improvements of an average of more than 34% as compared to traditional EKF. Interesting work is described in [28] where a tele-presence robot is utilized to estimate the safety of the drivers and pedestrians.

Although numerous designs have been proposed to tackle the transmission, monitoring and control of TPR [7,8,10,18,19,20,21,22,23,24,25,26,27,28], these implementations have certain limitations. The user always controls TPR from remote locations that result in issues either for the transmission or control of TPR. In most cases, the existing tele-presence robot systems are proposed without considering the collision characteristics [7,10,19,21,22,24,26,28]. The average transmission delay could cause a collision with an object. Therefore, a collision prevention system should be considered during the design of the TPR. Robust control is also needed in remote areas to control the TPR where it faces steering in congested sites. Thus, the robust control of the omnidirectional implementation is an additional concern that affects the performance/efficiency of the robot. Therefore, a robust design and implementation to cope with the aforementioned issues is an open research problem.

### 1.3. Novelty and Contributions

The originality of this paper is the robust control of the omnidirectional movement of TPR and the integration of Web Real-Time Communication (WebRTC) with the Dynamic Domain Name System (DDNS) server for the efficient transmission of videos. The contributions to this work are as follows:

- We have presented a tele-presence robot to offer the efficient video transmissions by using a WebRTC. The corresponding details are given in Section 3.3.
- Designing of an online portal using a hypertext markup language (HTML) that can be accessed from anywhere in the world. Then, the integration of our designed portal with the proposed tele-presence robot is an additional contribution.
- Performance evaluation of the WebRTC for three different video resolutions, i.e., 320×280 , 820×460 , and 900×590 pixels.
- We have proposed a Tricon sensor algorithm for the Kalman Filter to improve the robustness of the collision avoidance.
- Simulation of the TPR with Kalman filter for the crucial paths/hurdles to estimate the parameters, i.e., velocity and position.
- Hardware implementation, testing of the TPR in real environment and performance comparison with simulated results.

The remainder of this paper is organized as follows: The mathematical structure of omnidirectional, DC motor and Kalman filter is presented in Section 2. Our proposed design of an omnidirectional tele-presence robot is described in Section 3. The simulation results are given in Section 4. The hardware implementations and testing of our proposed tele-presence robot is presented in Section 5. Finally, Section 6 concludes the paper.

## 2. Preliminaries

The fundamental mathematical models associated with TPR are as follows: (i) modeling of omnidirectional wheels and (ii) dynamical modeling of DC motor. The corresponding details for these two models are further described in Section 2.1 and Section 2.2, respectively.

### 2.1. Modeling of Omnidirectional Wheels

**Figure 1:**
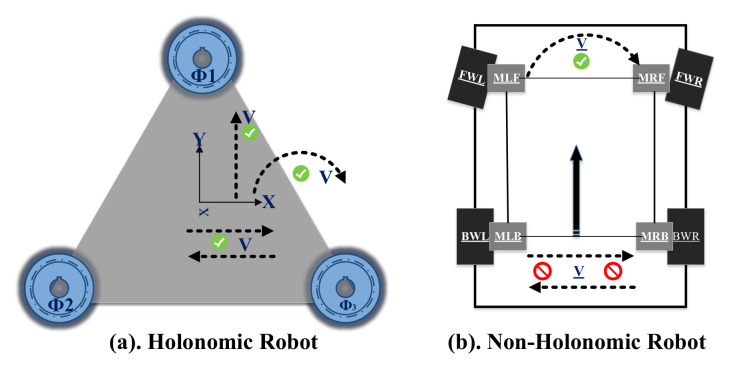
Difference between the motion of the Holonomic and Non-Holonomic robots.

The kinematic model of TPR includes two types of robots: (i) Holonomic and (ii) Non-Holonomic. The prior can move in any direction at any angle while the former can not move instantly in any direction. Moreover, the Non-Holonomic robots require some turning radius (steering angle) and time to turn in a specific direction. This type of robot needs parallel motion to turn in any direction. For example, car tires will move parallel to turn right or left. Therefore, Figure 1 shows the clear difference between the Holonomic and Non-Holonimic robots. It is important to note that we have used three wheels in the omnidirectional Holonomic system to obtain the rotation of the robot with zero turning radius.

**Figure 2:**
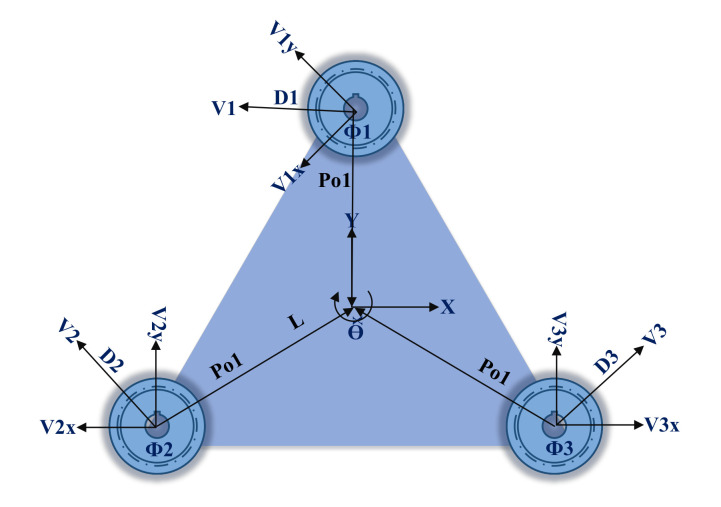
Kinematic model of the omnidirectional Holonomic robot.

The Kinematic model of the Holonomic robot is shown in Figure 2. It provides three degrees of freedom and it can pose all three directives: *x*, *y* and θ . In our design, we have used Kiwi omnidirectional design where three omnidirectional wheels are mounted symmetrically at 120-degree angles and can move in all directions. Each wheel moves with the angular moment of the DC gear motors. The symmetrical distance between the center and wheel is represented as *L*. The center of gravity of the robot coincides with the center of the local frame. Moreover, three omnidirectional wheels are located with symmetrical angles of 120, as shown in Figure 2. The center of mass of TPR is represented with O, the vector connecting O to the origin is denoted with $Po_{i}$ (*i* shows the number of wheel) and $V_{i}x,y$ is the direction vector of each omni wheel. The unitary rotation matrix, i.e., R(θ) , of TPR is given in Equation (1). Moreover, the vector connecting with the origin is shown in Equation (2).

(1) $$\begin{array}{c} R\left(\theta \right)=\left[\begin{array}{cc} cos(\theta ) & -sin(\theta )\\ sin(\theta ) & cos(\theta ) \end{array}\right] \end{array}$$

(2) $$\begin{array}{c} P_{o}i=\left[\begin{array}{c} x_{i}\\ y_{i} \end{array}\right]=R\left(\theta \right)L\left[\begin{array}{c} 0\\ 1 \end{array}\right] \end{array}$$

Using Equations (1) and (2), we have obtained $P_{o}1=L\left[\begin{array}{c} 0\\ 1 \end{array}\right]$ , $P_{o}2=\frac{L}{2}\left[\begin{array}{c} -1\\ -\sqrt{3} \end{array}\right]$ and $P_{o}3=\frac{L}{2}\left[\begin{array}{c} \sqrt{3}\\ -1 \end{array}\right]$ . Similarly, the drive direction of each wheel is $D_{1}=\left[\begin{array}{c} -1\\ 0 \end{array}\right]$ , $D_{2}=\frac{1}{2}\left[\begin{array}{c} \sqrt{3}\\ -1 \end{array}\right]$ and $D_{3}=\frac{1}{2}\left[\begin{array}{c} 1\\ \sqrt{3} \end{array}\right]$ . The velocity and position of TPR with respect to the global frame is $R_{i}=P_{o}+R\left(\theta +\frac{2\pi }{3}\left(i-1\right)\right)P_{o}i$ and $V_{i}=\dot{P_{o}}+\dot{R}\left(\theta +\frac{2\pi }{3}\left(i-1\right)\right)P_{o}i$ . The general form of a velocity matrix is given in Equation (3). Then, by substituting the aforementioned equations, we can obtain the velocity matrix as given in Equation (4).

(3) $$\begin{array}{c} \left[\begin{array}{c} V1\\ V2\\ V3 \end{array}\right]=P\left(\theta \right)\left[\begin{array}{c} \dot{x}\\ \dot{y}\\ \dot{\theta } \end{array}\right] \end{array}$$

(4) $$\begin{array}{c} P\left(\theta \right)=\left[\begin{array}{ccc} -cos(\theta ) & -sin(\theta ) & L\\ cos(\frac{\pi }{3}-\theta ) & sin(\frac{\pi }{3}-\theta ) & L\\ cos(\frac{\pi }{3}+\theta ) & sin(\frac{\pi }{3}+\theta ) & L \end{array}\right] \end{array}$$

The inverse kinematics for P(θ) is always singular for any value of θ . Then, the general form of the inverse kinematic matrix is illustrated in Equation (5). Finally, the inverse kinematic matrix is shown in Equation (6).

(5) $$\begin{array}{c} \left[\begin{array}{c} \dot{x}\\ \dot{y}\\ \dot{\theta } \end{array}\right]=P{\left(\theta \right)}^{-1}\left[\begin{array}{c} V1\\ V2\\ V3 \end{array}\right] \end{array}$$

(6) $$\begin{array}{c} P{\left(\theta \right)}^{-1}=\left[\begin{array}{ccc} -\frac{2}{3}cos\left(\theta \right) & \frac{2}{3}cos\left(\frac{\pi }{3}-\theta \right) & \frac{2}{3}cos\left(\frac{\pi }{3}+\theta \right)\\ -\frac{2}{3}sin\left(\theta \right) & \frac{2}{3}sin\left(\frac{\pi }{3}-\theta \right) & \frac{2}{3}sin\left(\frac{\pi }{3}+\theta \right)\\ \frac{1}{3L} & \frac{1}{3L} & \frac{1}{3L} \end{array}\right] \end{array}$$

For complete mathematical descriptions/derivations of the aforementioned equations, we refer readers to [29].

### 2.2. DC Motor Modeling

**Figure 3:**
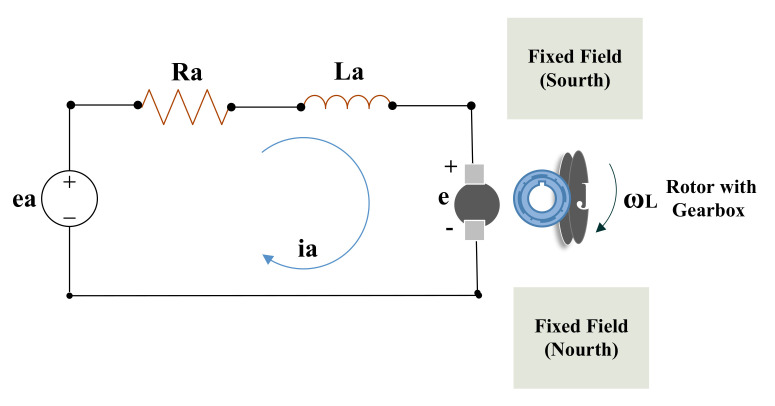
The schematic diagram of the DC motor.

The DC motor (an electromechanical component) is responsible for the movement of the robot. In order to analyze and receive angular velocity, the modeling of the electromechanical component is perceived. Therefore, in this work, the rotating coil with a fixed field has been included in the DC gear motor. The schematic diagram of the DC motor is represented in Figure 3. The corresponding mathematical equations of the DC motor are completely described in [30], and the input and output of the state-space model are represented in Equations (7) and (8), respectively.

(7) $$\begin{array}{c} \left[\begin{array}{c} \hat{\omega_{L}}\\ \hat{\theta_{L}}\\ \hat{i_{a}} \end{array}\right]=\left[\begin{array}{ccc} \frac{-D_{e}}{J_{e}} & 0 & \frac{-N_{1}K_{t}}{N_{2}J_{e}}\\ 1 & 0 & 0\\ \frac{-N_{2}K_{b}}{N_{1}L_{a}} & 0 & \frac{-R_{a}}{L_{a}} \end{array}\right]X+\left[\begin{array}{c} 0\\ 0\\ 1 \end{array}\right]e_{a} \end{array}$$

(8) $$\begin{array}{c} Y=\left[\begin{array}{ccc} \frac{N_{2}}{N_{1}} & 0 & 0 \end{array}\right] \end{array}$$

Rotation of DC motor [30,31]. The relation between angular and linear velocity can be presented by using $v=r\omega_{L}$ . The $\omega_{L}$ is the load shaft angular velocity (as shown in Figure 3) with its gear ratio, and the angular velocity of the motor is $\omega_{m}$ . The gear-motor is possessed by TPR, so we are using gear ratio with the help of Equations (9)–(12). The DC-motor constants calculation is presented in Table 1.

(9) $$\begin{array}{c} \frac{K_{t}}{R_{a}}=\frac{T_{stall}}{e_{a}} \end{array}$$

(10) $$\begin{array}{c} K_{b}=\frac{e_{a}}{\omega_{zero-load}} \end{array}$$

(11) $$\begin{array}{c} T=\omega_{zero-load}D_{e} \end{array}$$

(12) $$\begin{array}{c} J_{e}=\frac{T}{v\omega_{zero-load}^{2}} \end{array}$$

Consequently, we have calculated the constants, listed in Table 1, by using Equations (9)–(12) and the measured values of $R_{a}$ , $L_{a}$ .

### 2.3. Kalman Filter

The application(s) of the Kalman filter includes many areas of engineering, especially in signal processing, robotics, and embedded system. It is an iterative method to estimate the desired state of the dynamical system along with the noise. The essential feature of the Kalman filter is that it simultaneously performs the estimation, detection and coefficients update [32]. The state vector to estimate the position and velocity is represented in Equation (13).

(13) $$\begin{array}{c} \left[\begin{array}{c} P_{osT}\\ V_{elT} \end{array}\right]=\left[\begin{array}{cc} 1 & \Delta T\\ 0 & 1 \end{array}\right]\left[\begin{array}{c} P_{os.T-1}\\ V_{el..T-1} \end{array}\right]+\left[\begin{array}{c} \frac{\Delta T^{2}}{2}\\ \Delta T \end{array}\right]\left[\begin{array}{c} U_{T} \end{array}\right] \end{array}$$

In Equation (13), $P_{osT}$ , $V_{elT}$ and $U_{T}$ present the current position, velocity and acceleration of the TPR. For Equation (13), the prediction equation for Kalman filter is $\bar{X_{T}}=A{\hat{X}}_{T-1}+BU_{T}$ . The prediction for the current stage is based on the previous data. This predicts the coming stage along with the update and is performed with the incoming data from the used ultrasonic sensor. The output of the Kalman filter in terms of the position is $\bar{Z_{T}}=\left[\begin{array}{cc} 1 & 0 \end{array}\right]\left[\begin{array}{c} P_{osT}\\ V_{elT} \end{array}\right]$ , $\bar{Z_{T}}=C\hat{X_{T}}$ . Two terms, i.e., $\hat{X_{T}}$ , and $\bar{X_{T}}$ , are the estimated and predicted state vectors. Using the preceding equations, an updated TPR formulation is constructed in Equation (14).

(14) $$\begin{array}{c} \hat{X_{T}}=\bar{X_{T}}+K\left(Z_{T}-\bar{Z_{T}}\right) \end{array}$$

We will obtain the input from the sensors represented as *Z* in the update equation where $\bar{Z_{T}}$ is the estimated sensor measurements. In Equation (14), the Kalman gain is multiplied with the correction term. The gain of the Kalman filter is given in Equation (15). The compelete mathematical model is given in [33]. Equations (16) and (17) represent the prediction and covariance matrices.

(15) $$\begin{array}{c} K=\bar{P_{T}}C^{T}S^{-1} \end{array}$$

where

(16) $$\begin{array}{c} \bar{P_{T}}=\frac{COV(X_{T}-\bar{X_{T}})}{1-K^{T}C^{T}-KC+KSK^{T}} \end{array}$$

(17) $$\begin{array}{c} S=C\bar{P_{T}}C^{T}+COV\left(SensorNoise\right) \end{array}$$

## 3. Proposed Design of Omnidirectional Tele-Presence Robot

The complete design of our proposed omnidirectional tele-presence robot consists of (i) Tricon ultrasonic sensors, (ii) Kalman filter implementation and control and (iii) integration of our developed WebRTC-based application with the omnidirectional tele-presence robot for video transmission. The corresponding details for these blocks of our TPR design are given in Section 3.1–Section 3.3.

### 3.1. Our Proposed Algorithm for Tricon Ultrasonic Sensors

**Figure 4:**
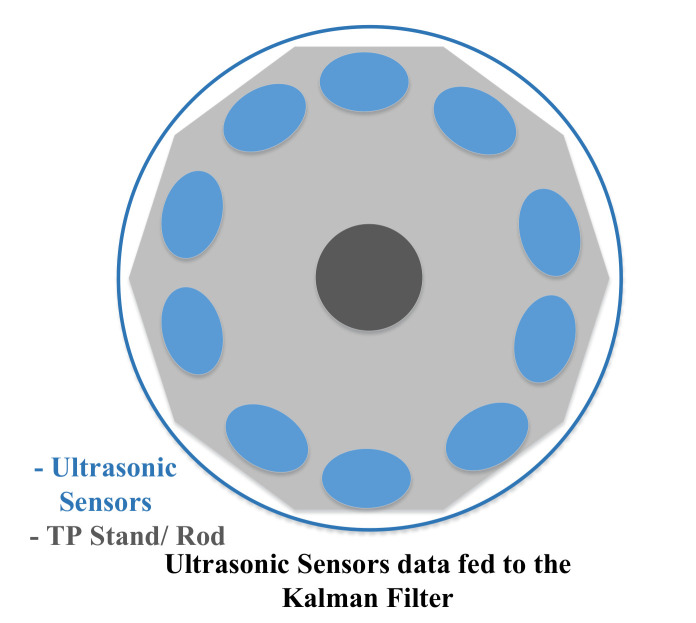
Ten ultrasonic sensors mounted in a circular order.

Our TPR offers omnidirectional drive; therefore, it is essential to cover protection with 360 degrees. It requires mounting different sensors on multiple angles to convert the collision space in the congested area. These sensors increase noise and establish delay penalties. The Kalman filter takes the sensor data and continues its process to predict and update the state of the TPR. So, we have to add multiple ultrasonic sensors in a circular way to cover the minimum possible angle of the sensor detection. To get the best approximation, we need the maximum number of ultrasonic sensors. Let us consider the ten sensors, as shown in Figure 4. These sensors are mounted in a circular way to the stand of the TPR. The ultrasonic sensors have a decagon shape, and the angle between the two sensors is 36 degrees. The sensors are responsible for taking the input from the physical environment, converting it into an analog signal, and then, the Kalman filter estimates the position of the TPR. As we increase the ultrasonic sensors, then the angle between the two sensors will decrease gradually, and the Kalman filter will give the best approximation for the position.

**Figure 5:**
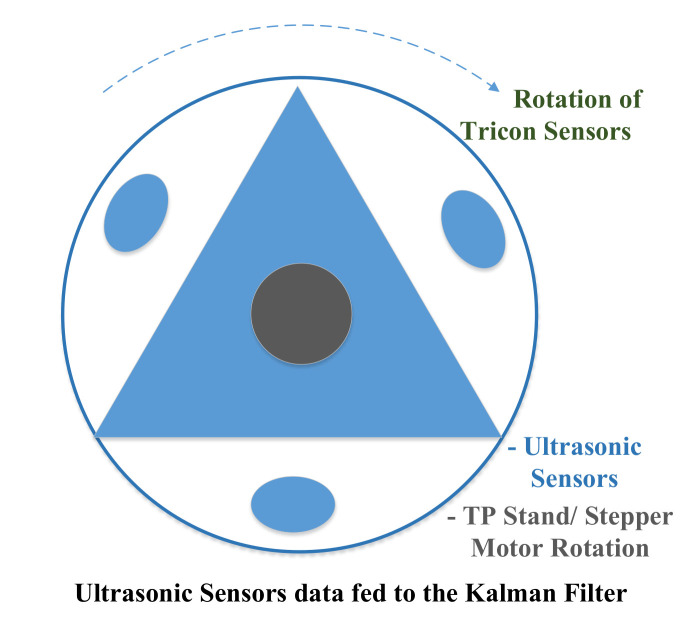
Tricon (three) ultrasonic sensors mounted in circular order.

**Figure 6:**
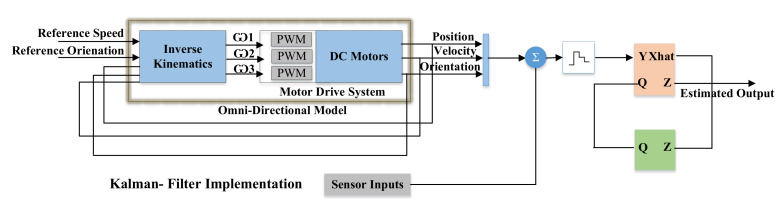
Architecture of robot kinematics and integration of Kalman filter in TPR.

In Figure 5, the sensors are mounted on the circular shaped-aluminum ring. Moreover, the stepper motor is connected to the center of the circular ring. The stepper motor rotates the ring, and physical data are recorded by these three sensors. The complete process of three sensors for the Kalman filter is illustrated in Algorithm 1. Therefore, to reduce the number of sensors and provide a sensor data for robust approximation, we proposed an Algorithm 1 to cover 360 degrees with three ultrasonic sensors that result in a robust implementation of Kalman filter. The Kalman filter will be described later in Section 3.2. The output of Algorithm 1 is input to the Kalman filter as we shown in Figure 6. The Tricon sensor algorithm facilitates the Kalman filter to obtain a better estimation of the velocity and position.
**Algorithm 1** Proposed Tricon algorithm for the ultrasonic sensors.

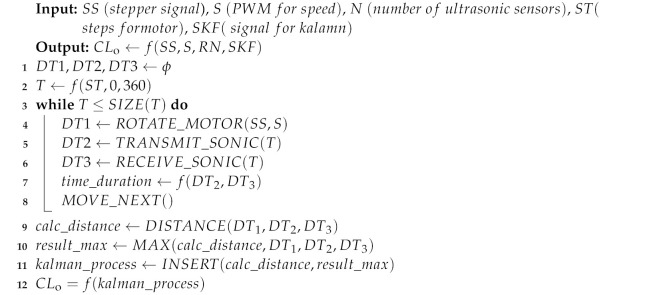



Algorithm 1 only uses three sensors, which are mounted at an angle of 120 degrees. That is why we call these sensors “Tricon sensors”. These sensors record the data from the physical environment and convert it into an electrical signal. The stepper motor at the central rod of the TPR rotate these three sensors, and the rotation depends on the speed of the TPR. As the speed of the TPR increases, then the speed of these three ultrasonic sensors also increases to record the maximum values (samples/ms) of the ultrasonic signal. The higher the recorded values, the higher the probability of the estimation for the predictor in the Kalman filter. The recorded values of ultrasonic signals are then passed sequentially to the Kalman filter; this all happens at a very high processing speed. The processing speed depends upon the clock rate of the microprocessor of the controller. Furthermore, Algorithm 1 illustrates the computation for one rotation of ultrasonic sensors. It initialized the empty arrays for the intermediate computation, and then, it calculates the timing samples for 360 degrees of the rotation. The loop calculates the output of the functions. It rotates the motor; then, it transmits and receives ultrasonic signals. It calculates time duration, and then, it moves for the next timing instance. It also calculates the distance between a robot and an obstacle. Finally, it retrieves the maximum signals and sends them to the Kalman filter along with the distance.

### 3.2. Control and Integration of Kalman Filter in TPR

In Figure 6, the light orange block is the Kalman filter. The Kalman filter estimates the position of the TPR according to the input of the ultrasonic sensors. There is a two-step for the working of the Kalman filter to estimate the next position of the TPR. Initially, it predicts the state based on the previous state and sensor data. Then, it updates the state of the TPR in terms of position. There are two variables, which are reference speed and orientation. These are the inputs to the control system or set points for the output variables. This represents the desired operating value of the output, i.e., position, velocity and orientation. The inverse kinematics consists of a state-space model which generates angular velocities. Based on the angular velocities, the PWM (pulse-width modulation) signals are generated for the speed of the DC gear motors. We integrate and tune the famous PID controller (a famous type of a controller) to control the speed of the DC gear motors [34]. In the next step, position, velocity and orientation vectors are fed to Zero-Order Hold (ZOH) for the practical signal reconstruction. It reconstructs the signal to be used in the Kalman filter for the state estimation, i.e., *Z* (that represent the error covariance and estimate output/state). The block with green color performs time-varying process noise covariance.

As we summarized with reference to Figure 6, the reference position determines the position of an omnidirectional robot in X-Y plane. The omnidirectional model/system is represented to process the feedback parallel to the Kalman filter. The outputs of the omnidirectional system go to the Kalman filter, which includes the sensor inputs. The predictor estimates the next state whose output is Xhat (estimated vectors, i.e., position, velocity and orientation), and then, the updater modifies the state, whose output is *Z*. The PID controller is used for the smooth drive and control of gear motor [35]. In this way, the system keeps on working.

### 3.3. Webrtc Integration in the Tele-Presence Robot (TPR)

**Figure 7:**
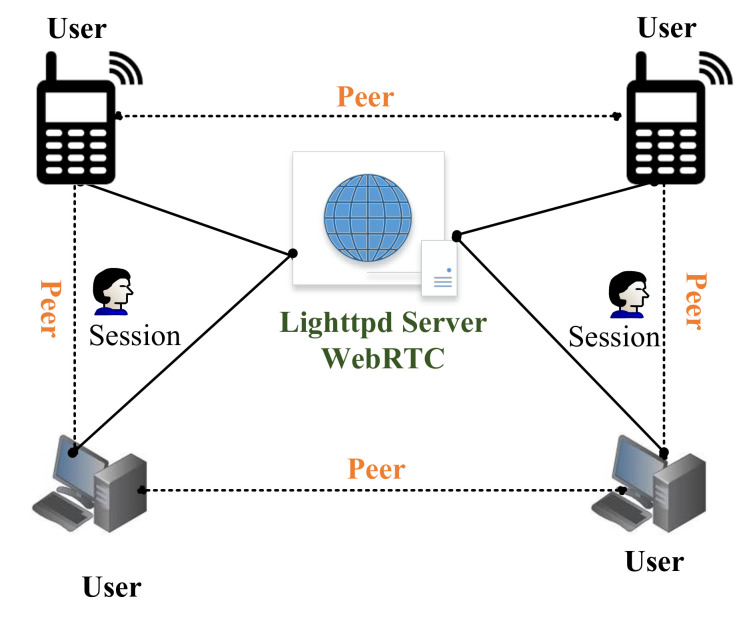
A generic overview for the integration of WebRTC in TPR.

Since developers are working on the full-duplex communication between peers and the server to peer, the enhanced version of a full-duplex is real-time communication. WebRTC is an example of such communication which is based on java, CSS, JS and HTML. WebRTC is open-source developed and released by Google [36]. We have used WebRTC to provide the video and conference video between the persons. This API is based on javascript to support the communication between web and mobile or web to web. WebRTC is implemented using a Lighttpd web server, which runs on Raspberry Pi 3 [37]. Lighttpd is a fast and reliable web server, which supports PHP, SQL, HTTP redirect, Ipv4, and Ipv6 compatibility with parallel connections. Therefore, in our work, we have installed a Lighttpd web server on Raspberry Pi. Then, the WebRTC is implemented on the installed server, as shown in Figure 7.

Figure 7 reveals that the users are connected in bi-directional mode. Moreover, we have used Javascript APIs to process the required conference session. MediaStream API is used to access the camera, microphone, light and speakers. The MediaStream input/output API synchronizes the data between two nodes of the real-time connection. The getUserMedia() is a javascript function that is used to send call requests on another node of the communication. The attachMediaStream() function is used to acknowledge the requested call on the node of the communication. RTCPeerConnection defines the peer-to-peer connection between the users. Furthermore, it handles all the stream bytes and streaming of the real-time video [38]. The DataChannel interface captures the incoming and outgoing data of the streaming video from the peer-to-peer connection. The signaling stage handles all the notifications and control of the real-time communication. Signaling is implemented over Extensible Messaging and Presence Protocol (XMPP), which controls all the progress such as stop, accept, reject, mute, unmute, etc. Consequently, the javascript is integrated with HTML to get the display window of the chat and conference of the TPR. The javascript is also responsible for transmitting the control signals to a local server for the movement of the TPR. In addition, the filters, recording mechanism and other video features are implemented to create a user-friendly environment.

**Figure 8:**
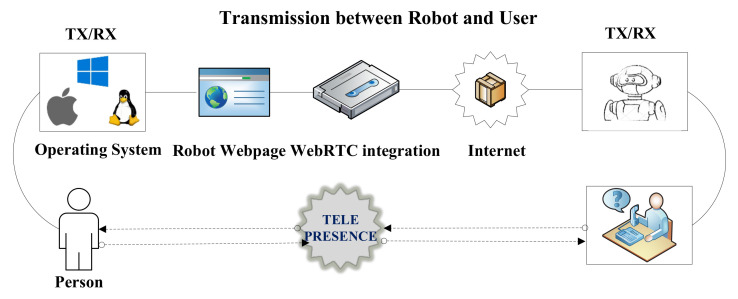
Complete sequence of WebRTC, Javascript signalizing API implementation.

The complete sequence of WebRTC and javascript signaling API implementation is illustrated in Figure 8. The communication between the observer and presenter is shown as full-duplex where the sequence of packets traveled through the internet. We have designed a dedicated portal to control the TPR. Each user has their login and password to access the portal. The user can open this portal to any device, e.g., PC (Windows/Apple) or mobile. After login, the page will show the integration of WebRTC for real-time video streaming, and it also includes the control panel to control the TPR. Finally, the user and person sitting in a remote area can chat with each other.

## 4. Simulation Results

This section describes the simulation results for the WebRTC and control of omnidirectional movement of the robot.

### 4.1. Performance Evaluation of Webrtc

The performance evaluation results of WebRTC are given in Table 2. Column one provides the used API. Columns two to five provide the factors that affect the data transmission performance, i.e., resolution of the transmitted video(s), time of the encoded video, average transmission delay and throughput. We have used three different cases with different video resolutions, i.e., 320×280 , 820×460 and 900×590 . The throughput is calculated by using Equation (18).

(18) $$\;\mathrm{Throughput}\;=\frac{L}{d/V+L/B}$$

In Equation (18), *L* shows the packet length to be transmitted (in bits), $\frac{d}{V}$ is the sum of the propagation delay and $\frac{L}{B}$ is the actual transmission time (or the amount of time devoted to that packet).

Table 2 shows that the average encoding time is increased when processing higher-resolution videos. Delay during transmission may occur which in turn will result in increasing buffer size for temporary storage, causing longer latency. However, a higher resolution results in more data to be transmitted but does not affect the throughput of the streaming due to congestion control.

### 4.2. Simulation of Controller along with Kalman Filter

**Figure 9:**
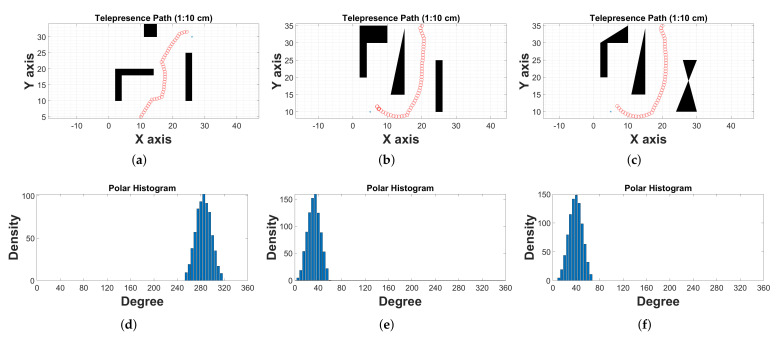
(**a**) Case 1: square, L and I shape hurdles; (**b**) Case 2: polygon, I and triangular hurdles; (**c**) Case 3: square, L and I shape hurdles; (**d**) Case 1: Histogram of tricon sensors; (**e**) Case 2: Histogram of tricon sensors; (**f**) Case 3: Histogram of tricon sensors.

We have simulated the controller as defined in Section 3.2. Moreover, we have simulated the model, which is illustrated in Figure 6. The inverse kinematics block is inferred to generate angular velocity for each motor, which is further used to generate PWM signal for the corresponding motor. Therefore, Figure 9 shows three different cases for the given path. The desired path is the set of coordinates in the X-Y plane, starting point ( $x_{1},y_{1}$ ) and the destination point ( $x_{2},y_{2}$ ). We have provided instructions/commands through the internet to move the TPR. During simulation, we have created a module that behaves as a path generator. Figure 9a–c contain three hurdles of different types and coordinates, respectively. Similarly, panel Figure 9c represents a similar type of placement for hurdles but with an uneven surface. The panels Figure 9d–f illustrate the histogram for the response of Tricon ultrasonic sensors. The x-axis of the panels Figure 9a–c shows the displacement in the x-direction and the y-axis represents the displacement in the y-direction. We scale down from 1 m to 10 cm for simplicity. Similarly, the x-axis of the panels Figure 9d–f shows the angle in degrees and the y-axis shows the density. Considering panels Figure 9a,d, the robot is oriented toward 290 degrees because the density is maximum for this point. The difference between case-2 and case-3 is to analyze the effect of uneven surface for Tricon sensor arrangement. From the density histogram, we can see the maximum peak of the density, but it is spread between zero and sixty.

**Figure 10:**
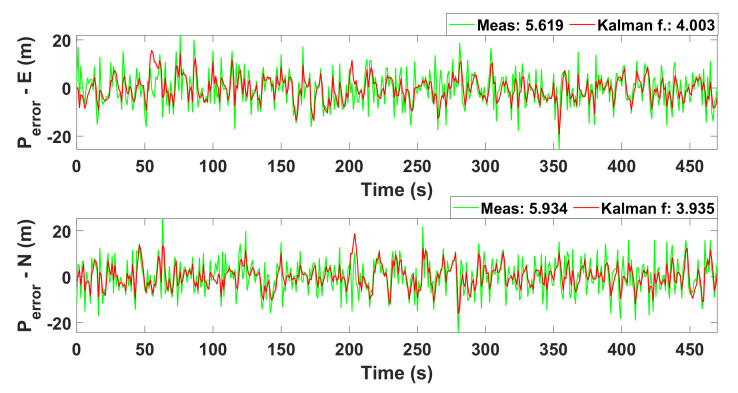
Simulation of Kalman filter for autonomous drive.

**Figure 11:**
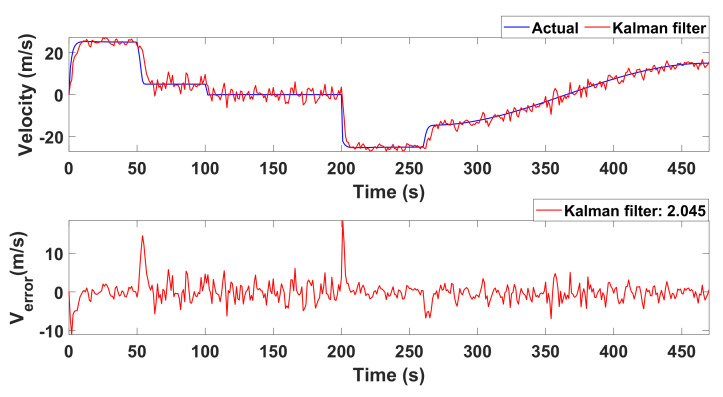
Simulation of the Kalman filter for velocity and position estimation.

The simulated results for Kalman filter are illustrated in Figure 10 and Figure 11, respectively. Figure 10 provides the simulation of Kalman filter for autonomous drive. Similarly, Figure 11 provides the simulation of the Kalman filter for velocity and position estimation. In both figures, the x-axis represents the time in seconds. Similarly, the y-axis in Figure 10 shows the position error for the east and north (x and y directions) directions. The y-axis in Figure 11 shows velocity (see the top panel) and the error (see the lower panel) in the linear velocity of the robot.

Figure 10 reveals that the error between the measured and estimated positions is 27%, which is lower than the measured data for sensors. It reduces the error during the decision of collision protection. Figure 11 represents the actual and estimated combined velocity of three wheels. We can analyze that the velocity is maximum for two instances of time. This is the case when a robot is turning to avoid obstacles. From the figures, it is clear that the mean error values for the estimation of position and velocity are 5.77% and 2.04%.

## 5. Hardware Demonstrations and Testing of Tpr

The corresponding hardware demonstration and testing of our TPR is presented in Section 5.1 and Section 5.2, respectively. Moreover, the significance of this work is highlighted in Section 5.3.

### 5.1. Hardware Demonstration

TPR has been implemented by using three omnidirectional wheels associated with the motors, and those wheels are affirmed with the same output signal that helps in driving the motor in a triangular shape. In this way, omni-wheels simultaneously move. The TPR is implemented by using the components presented in Table 3. To stabilize the speed of the utilized motors, the PID controller is used along with the omnidirectional control, as used in [34].

This includes Raspberry Pi 3 and is programmed with compatible language. The Raspberry Pi is acting as a server for the hosting of the control panel. The designed HTML page is stored in the memory of the Raspberry Pi; when a user makes a request(s)—then the DNS is responsible for forwarding the request to the router and the router forwards it to the Raspberry Pi. The Raspberry Pi activates the script according to the instructions of the user. The ultrasonic sensors are responsible for reading the data. The Analog-to-Digital (ATD) converter effectively converts the data in a digital form. The Raspberry Pi reads the digital data and processes it through the proposed Tricon algorithm. After effective processing of the data, micropossessors provide PWM signals to ensure the motor drives seamlessly.

**Figure 12:**
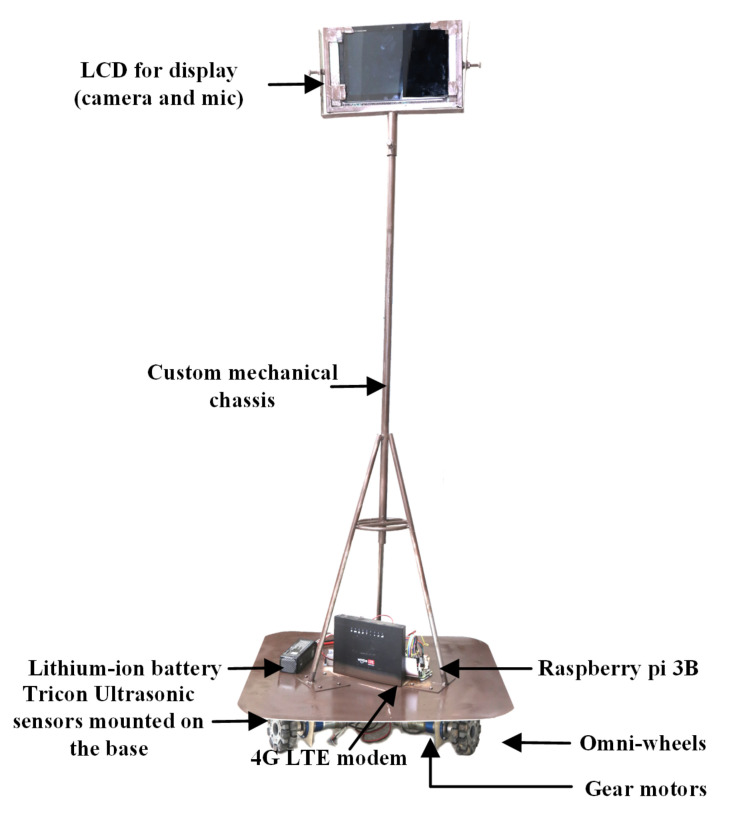
Final hardware demonstration of tele-presence robot (TPR).

The hardware implementation also includes the reception of the digital video signal and control signal. Both signals travel through the internet. The video signals decode through webRTC and webserver. Generally, this section requires the control signal implementation. The received control signal goes to the microprocessor (Raspberry Pi), and then the program gives instructions to the motor driver according to the received instruction. A motor driver moves the omnidirectional drive system and the robot moves finally. The complete snippet of the implemented robot is presented in Figure 12.

### 5.2. Testing of the Tele-Presence Robot

**Figure 13:**
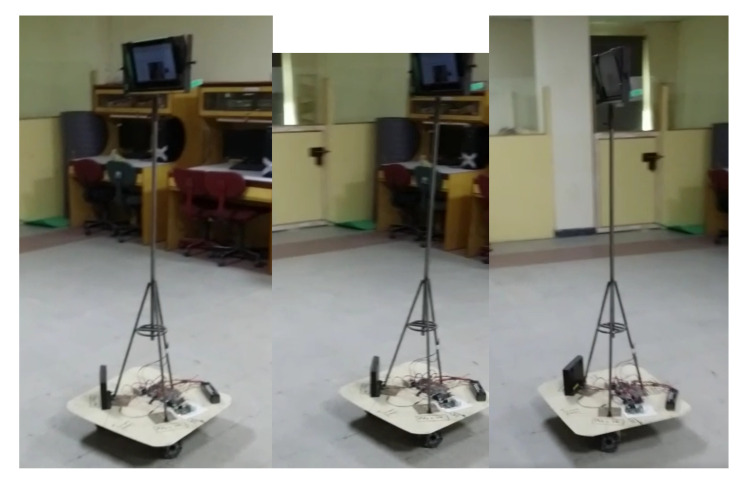
Working of robot during the testing from a remote location.

**Figure 14:**
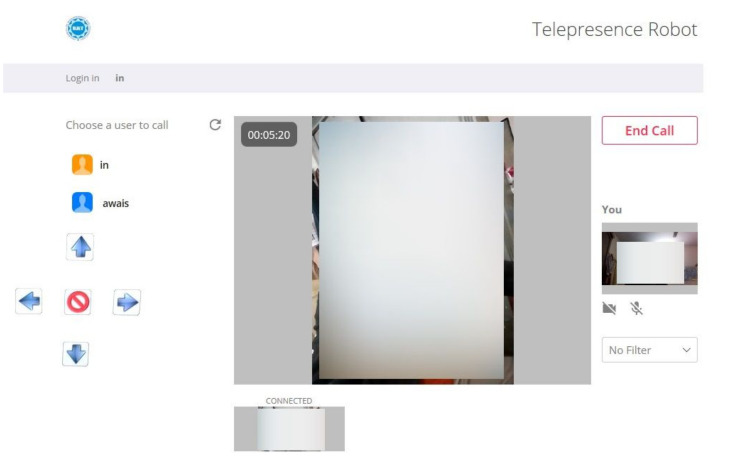
Final testing of tele-presence robot (TPR).

The designed robot was tested to verify the performance and feasibility on the 4G internet. The robot was placed in the Digital Logic Lab (Location A as illustrated in Figure 13) and tested from the Projects Lab (Location B). Both locations (Location A and B) consist of numerous hurdles, and we evaluated the performance of the robot. The hurdles include tables, chairs and triangular corners in the lab due to the structure. The area of both labs is 900 m ${}^{2}$ ; thus, an operator was connected to the remote network. The robot was connected with the 4G Long-Term Evolution (LTE) router, and the client (Laptop) was connected to the Local Area Network (LAN) internet. We have used the accelerometer to measure the current position of the robot; the error between the measured and estimated position of the robot is illustrated in Figure 10. We have used an encoder to measure the velocity of the robot. This is actual velocity; the graph of the actual velocity and estimated velocity is given in Figure 11. There is a total of six buttons in the control panel, as shown in Figure 14. These buttons are required to move the robot in different directions, i.e., right, left, forward, backward, clockwise 360 degrees rotation and anti-clockwise 360 degrees rotation.

**Figure 15:**
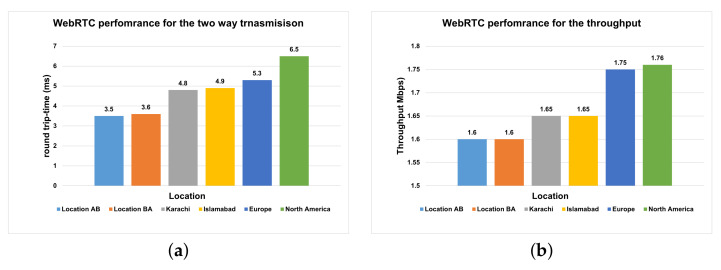
(**a**) Round transmission time (ms) for multiple locations; (**b**) throughput (kbps) with respect to multiple locations.

We switched webRTC to 320×280 and requested our volunteers from multiple locations to test the robot. For these locations, we measured the average delay and transmission time for the round trip. We created multiple usernames and passwords for the volunteers and asked them to operate our tele-presence robot. We create a simple function that captures the packets time and then measures the transmission time and throughput. This is considered real-time communication between the browser and tele-presence robot. By utilizing these values, we further analyzed the variation with respect to the targeted control location. Figure 15 illustrates the trade-off between the location, transmission time and throughput. The left panel shows that the transmission time is increasing as we increase the distance of the remote user. As we move far from the robot, the packets will travel from different routers and round-trip transmission time is increasing.

The right panel illustrates the throughput concerning the location. The throughput is increasing concerning the distance; thus, the performance of the transmission is decreasing. In comparison with the solution in [13], the average throughput of our solution is 1600 kbps, which is less. Moreover, the average delay and encoding time are also less compared to their solution. The round trip time varies between 3 and 20 ms with time. The aforementioned results are reported for 320. Our results are also reported for the same resolution; this justifies the fair comparison. It is noteworthy that the round-trip time of the solution is less than 400 ms [7]. In our solution, the delay fluctuates between 1 and 80 ms, which is far from the 400 ms, as given in [7]. The round-trip time of our experiments is significantly lower than [39]; their results revealed a 40 ms delay as a round-trip time. On the other hand, the connection time of our solution is also faster as compared to the Skype platform [40]. Moreover, the transmission rate of WebRTC operates the transmission on the available bandwidth of the network, whereas the Skype platform exploits the video resolution for the transmission [40]. More essentially, WebRTC is free. easily accessible and open source. The development in WebRTC technology can provide flexibility for further extension. Nevertheless, the performance also depends on the hardware of the system. The video quality also relies on the bandwidth of the internet connection.

### 5.3. Significance of the Proposed TPR

The main objective of this work was to design and implement a tele-presence robot for the use of medical purposes such as in hospitals. More precisely, in the hospital’s wards, if a patient needs an instant service when the physician is not physically available, then the proposed TPR could be used by the physician to facilitate the patients. Furthermore, we prefer to use a local network and server, where many physicians and patients can communicate with each other. Moreover, it could also be used in old age homes to enable the older residents to communicate with their beloved ones. More interestingly, the proposed TPR could also be used to conduct virtual classes to provide distance learning. In a nutshell, it could be used to address different social needs.

## 6. Conclusions and Future Work

This paper presents the design and implementation of the TPR. The robustness of the TPR is evaluated in the form of video transmission and omnidirectional control. For the transmission of video, our solution achieves a throughput of 1.60 Mbps and an average delay of 1.50 ms. We repeated the same experiment for higher resolutions. For the robust evaluation of the Kalman filter, we measured the real-time position and velocity and compared the results with simulated results. Our comparison for the Kalman filter confirms the reliable estimation of velocity and position with the support of our Tricon sensor algorithm. The tele-presence can cope with tight and tough obstacles such as acute angles, right angles, and the curvy path along with the inclined surface. These are all possible scenarios that we have considered concerning social applications. The number of sensors for the assessment of the Kalman filter plays an important role in the estimation. Our Tricon sensor algorithms aid the Kalman filter to improve the estimation with only three sensors. We tested our robot from multiple remote locations to confirm the robustness of our WebRTC implementation and Kalman filter. The testing session of tele-presence is evident to the efficiency and feasibility of the robot for several applications such as medical purposes and many other social needs. In future, we are going to add multiple medical instruments in the robot to capture the real time of the patients. On the other hand, we will also integrate the data of the patients with the database of the patients.

## Figures and Tables

**Table 1:** Specification for DC motor.

Constants	Description	Value
$D_{e}$	Equivalent viscous damping	1.2 × $10^{-6}$ Nm s/rad
$J_{e}$	Sum of the motor inertia	3.35 × $10^{-11}$ kg· m ${}^{2}$
$R_{a}$	Resistance of motor rotor	340.0 Ω
$K_{b}$	Armature constant	0.009540
$L_{a}$	Inductance of motor rotor	0.120 mH

**Table 2:** Performance evaluation of WebRTC.

API	Resolution	Encode (ms)	Avg. Delay (ms)	Throughput (Mbps)
WebRTC	320 × 280	2.00±0.1	1.50±0.1	1.60±0.1
WebRTC	820 × 460	3.00±0.1	2.00±0.1	1.55±0.1
WebRTC	900 × 590	3.50±0.1	2.70±0.1	1.52±0.1

**Table 3:** Components of TPR.

Components	Rating/Model/Value
Chassis	16 guage Alloy
Microprocessor	Raspberry pi Model B
Ultrasonic sensors	1.2 V
Accelerometer	MPU6050
Battery (Li-po)	11.40 V 6A
Motor-Driver	BTN7971
Motors (DC-Gear) with encoders	FAULHABER 3557K012C
Router	DNS/Internet connectivity
Omnidirectional wheels	3-way

## Data Availability

Not applicable.
